# ‘Unforeseeable’ and ‘inevitable’: Constructions of prison suicide in Scotland’s Fatal Accidents and Sudden Deaths Inquiries

**DOI:** 10.1177/17488958241252954

**Published:** 2024-05-06

**Authors:** Sarah I Huque, Rebecca Helman, Joe Anderson, Amy Chandler

**Affiliations:** The University of Edinburgh, UK

**Keywords:** Qualitative analysis, Scottish prisons, suicide reviews

## Abstract

Rates of suicides are on the rise in Scottish prisons. Fatal Accident and Sudden Deaths Inquiries (FAIs) carried out by sheriffs following these deaths provide a valuable source of knowledge regarding how the criminal justice system understands and responds to incarcerated people’s suicides. Informed by literature in critical suicide studies and narrative criminology, we conducted an abductive, narrative qualitative analysis of 37 FAI reports of incarcerated people’s suicides published between 2016 and 2021, in Scotland. We argue that the FAIs explicitly individualise incarcerated people’s suicides, deploying explanatory narratives of (1) mental illness, (2) social history and (3) problematic substance use, while (4) simultaneously emphasising and undermining incarcerated people’s testimony and agency. By conceptualising suicide as paradoxically ‘unforeseeable’ and ‘inevitable’, these narratives shift blame onto the individual while absolving the prison system. Our analysis contributes towards understanding of how institutional procedures are implicated in social scripts and practices around suicide in Scottish prisons.

## Introduction

There is considerable debate about key drivers of suicides in prisons. A large body of research highlights relationships between rates of mental illness or distress and substance use among incarcerated people and high rates of suicide within prisons (Bird, 2008; Duke et al., 2023; Gilling-McIntosh et al., 2022; Simms et al., 2019; Tweed et al., 2021). Psychological and psychiatric perspectives often frame mental illness and substance use as ‘imported’ risks, vulnerabilities or pathologies that incarcerated people ‘bring with them’ into prison (Armstrong and McGhee, 2019; Mills and Kendall, 2018; Sim, 2018). However, critical perspectives highlight how prison contexts themselves compound and produce various forms of distress, including suicidality (Armstrong and McGhee, 2019; Wilson et al., 2020). As Sim (2018) argued, focusing on ‘individual vulnerabilities’ as causes of suicides in prisons ‘distracts attention away from the destructive, structural operationalisation of power inside’ (p. 244). In this article, we explore these explanatory tensions, focusing on suicides in Scottish prisons.

### Prison suicide in Scotland

There are approximately 7500 people incarcerated in Scotland, which, as of September 2023, represents 162 per 100,000 people (ScotPHO, 2023; Sturge and Carthew, 2023). Research indicates that rates of both incarceration and suicide in prison are higher than those in England and Wales and other European countries, with evidence of a further rise in suicides in recent years (Armstrong et al., 2022; Bird, 2020; Fazel et al., 2017).

Existing scholarship on mental health care in Scottish prisons identifies shortcomings in the culture of treatment, including a reluctance by incarcerated people to share experiences of mental ill health or distress because of a lack of compassion and respect from guards (Gilling-McIntosh et al., 2022). Issues of disclosure are particularly important for people experiencing suicidality. Tomczak (2018) highlights that ‘prison cultures may position prisoners as requiring suspicion and being unworthy of health and care’ (p. 28). Similarly, Barkas et al. (2021a) and Armstrong et al. (2022) highlight the lack of comprehensive, timely care afforded to people in prison, potentially contributing to otherwise preventable deaths. The inability of incarcerated people to manage and improve their own health within this system also generates feelings of hopelessness that may drive both substance misuse and suicidality (Fraser, 2021).

Specific policies have been developed to address suicides in Scottish prisons. Most recently, the Talk to Me Strategy (TTM), replacing the previous ACT2Care strategy, was adopted as the official suicide-prevention strategy of the Scottish Prison Service (SPS) in 2016. According to the SPS (2016), the aims of TTM are to ‘assume a shared responsibility for the care of those “at risk” of suicide’, ‘provide a person-centred care pathway based on an individual’s needs, strengths and assets’ and develop an ‘environment where people in our custody can ask for help’ (p. 4). Since the introduction of TTM in 2016, there has been a 42% rise in the number of suicides in Scottish Prisons (Armstrong et al., 2022), raising questions about whether the policy is adequately responding to this issue. Within this context, it is important to explore how prison suicides are understood and how these understandings inform suicide-prevention strategies. One way of approaching this is through the analysis of the official reporting of suicide deaths.

In Scotland, sheriffs conduct a Fatal Accidents and Sudden Deaths Inquiry (FAI) of every prison suicide. FAIs are the primary way in which the procedural requirements of the right to life in relation to deaths which occur in custody are addressed in Scotland, in line with Article 2 of the European Convention on Human Rights (Sinclair-Gieben et al., 2021). This procedure is similar to the conducting of coronial inquests in England and Wales. The sheriff generates a report outlining the findings, considering both documents and witness testimony. The stated aims of the FAI process are establishing ‘the circumstances of the death’ and considering ‘what steps (if any) might be taken to prevent other deaths in similar circumstances’ (Inquiries into Fatal Accidents and Sudden Deaths etc. (Scotland) Act 2016, 2016, n.p.). FAIs are inquisitorial rather than adversarial procedures, which cannot determine legal culpability. The sheriff cannot make a finding of fault or apportion blame against people or institutions that might have contributed to the death. They are, however, intended to identify shortcomings and ways the prison system could have acted differently to prevent a particular suicide; as such, sheriffs can make findings of defect within prison systems. Our analysis indicates findings of defect are rare.

Reviews of deaths in prisons have been identified as an important but underexplored source of data, which can provide significant information about deaths and responses to deaths within prisons (Tomczak, 2022). Despite the potential for death reviews to promote accountability and improve health and safety in prisons, research in England and Wales has highlighted that these reviews focus narrowly on factual details, compliance with existing policy and procedure and case-by-case analysis, rather than engaging with systematic conditions that contribute to deaths in prisons (Aitken, 2022; Tomczak et al., 2023). This failure to engage with broader prison contexts has been identified as a barrier to identifying appropriate and meaningful recommendations for change (Shalev and Tomczak, 2023). While there is a growing body of research focusing on prison death reviews, including those related to deaths by suicide, in England and Wales, in Scotland FAIs have received limited scholarly attention.^1^ Further complicating the comparability of research into prison suicides between England and Wales and Scotland are differences in prison systems, procedures and publication of data between devolved nations.

In this article, we present a qualitative analysis of FAI reports of suicide deaths in Scottish prisons, examining the narratives used to construct suicidal individuals and the circumstances of their deaths. We find the FAIs lack consideration of how prison environments produce or exacerbate suicidality and highlight three explanatory narratives (mental illness, social history, substance misuse), which are deployed within FAIs to locate suicide as an issue with(in) incarcerated people and not the prison system. We argue that FAIs constitute a bureaucratic function in the wake of unexpected deaths in prison and do not adequately take account of the complex, institutionally embedded factors that might lead someone to attempt suicide.

By highlighting mental illness, substance misuse and social history as causes of suicide, the FAIs narratively construct individualised understandings of suicide, de-contextualising these deaths. These dominant explanatory narratives are underwritten by an ambiguous approach to incarcerated peoples’ agency. On the one hand, an individual’s statements about their suicidal thoughts and intentions (or lack thereof) are mobilised as authoritative statements of truth, while on the other, incarcerated peoples’ complaints about their care are undermined and discredited, effectively absolving prison systems of blame. This reflects a broader attitude towards incarcerated people as ‘social junk’ (Spitzer, 1975: 645), which reinforces the kinds of treatment and alienation that may cause suicidality within prisons (Armstrong et al., 2021).

### Critical suicide studies and narrative criminology

Our analysis of the FAIs is informed by scholarship in the fields of critical suicide studies and narrative criminology. Engaging both theoretical and methodological tools (Chandler et al., 2021), critical suicide studies questions often taken for granted understandings about suicide – for example, Marsh’s (2010) historical examination of how suicide came to be understood almost universally through a (bio)medical lens. A growing body of scholarship has developed critiques of approaches that focus narrowly on suicide as a sign of mental disorder (White et al., 2016). Critical suicide studies highlights the diversity of meanings that suicide can have, as well as the significant links between suicide and the social, cultural, political, and environmental contexts in which it takes place (Button and Marsh, 2020; Fitzpatrick, 2021; Mills, 2018; White, 2017).

A particular contribution of critical suicidology has been demonstrating how narrow conceptualisations of suicide can – perversely – problematically constrain suicide-prevention policies and practices (e.g. White et al., 2016). Similarly, our analysis identifies significant limitations in how suicide is understood within publicly available, official accounts, including how suicide is responded to within prisons.

Our analysis is grounded in and informed by narrative criminology (Fleetwood and Sandberg, 2021; Presser, 2022; Presser and Sandberg, 2019), which suggests that stories guide and motivate social behaviour, as well as delineating insiders and outsiders. Stories about incarcerated people produced by FAIs are constituted by specific institutional, political and social contexts, making their ‘accuracy’ less important than the particular narratives deployed to explain suicide and its causes. Narrative criminology emphasises that stories can legitimise responses, compel certain kinds of action and become bound up in affirming harmful practices (Presser, 2022). Our critical approach recognises that death reviews do not merely ‘discover’ existing facts but rather that these procedures *produce* particular facts about deaths in prison (Aitken, 2022). Informed by constructionist perspectives, our analysis thus seeks to critically interrogate the knowledge that is produced about suicide in prison to highlight the ‘legitimizing scripts and logics’ (Boutcher, 2017: 544) that are used to understand these deaths (Scourfield et al., 2012).

The highly formalised, legalistic method of storytelling utilised by sheriffs in FAIs indicates that the SPS seeks to distance itself from incarcerated peoples’ suicides, primarily pointing to factors outside of its control and building narratives that blame the individual or their life history for their actions. We argue that this narrative style and presentation can render suicides ‘unforeseeable’ and/or ‘inevitable’, located solely in the individual. For example, in one FAI, the sheriff states:

Suicide in prison, as in the wider community will never be eradicated. A person determined to take their own life will generally succeed … the suicide rate in prisons is about nine times that in the community … persons with significant social and health problems (physical, mental and psychological) are disproportionately represented in the prison population. However, the Scottish Prison Service operates a robust and comprehensive strategy designed to prevent or at least reduce the numbers of suicides in prison.

This sheriff highlights high rates of prison suicides as relating to factors imprisoned people ‘bring with them’, noting the intersectional marginalization often faced by those who have contact with prison systems, rather than focusing on the impact of prison as an institution. The sheriff also notes the ‘robustness’ of the SPS suicide-prevention strategy, even as numbers of suicides increase within Scottish prisons. Using narrative criminology to critically engage with stories and the motivations behind telling them, including those constructed within FAI reports, enables us to look at how what is written (and what is left out) might influence findings of fault on the part of the prison. This has implications for how suicides are responded to within prison contexts and for understandings of and responses to suicide in Scotland more broadly.

## Methods

This article draws on a narrative-informed thematic and abductive analytical approach (Timmermans and Tavory, 2022) of 37 FAI reports. Our inquiry focused on exploring how FAIs explained and made sense of suicides in prisons, what common and uncommon ways of understanding suicide were present and what patterns could be identified.

We downloaded all available FAI reports from the SPS’s website (n = 81). In 2016, the law regarding the process of conducting FAIs changed, coinciding with the implementation of the TTM suicide-prevention strategy; therefore, we excluded any FAI reports completed prior to 2016, leaving 38 reports. One further report was excluded due to an indeterminate finding in the death (n = 37). However, due to, at times, extensive delays in the process of conducting and releasing the FAI reports,^2^ some reports in our study involve deaths that occurred prior to 2016 (when the ACT2Care strategy was still in place).

Reports were coded using NVivo software; analysis was refined through team discussions around findings from coding. Reports were assigned numbers and identified using this number plus a prison identification code. Authors 1, 2 and 3 independently coded the same subset of the sample, in order to compare results and develop a consistent coding procedure. Following this, the full sample was randomly assigned to authors 1, 2 and 3 to read and conduct initial coding; reports from the same prison were grouped together to ensure continuity of researcher within said prison.

Authors 1, 2 and 3 each conducted an initial round of coding based on a list agreed by the full team, generated during initial readings of the reports and sub-set coding. All four authors then reviewed these codes, generating a new list for second-round coding based on analysis of emerging ideas and themes, raising the need for additional codes as the process continued. Consensus on what to do with any issues arising was determined through discussions between all authors.

We conducted an analysis of common tropes, ideas, and language used by sheriffs to describe the deceased person, the process of the inquiry and the factors that were determined to have contributed to the suicide. In this article, we present findings based around major themes of mental illness, substance use, social history and incarcerated peoples’ involvement in suicide prevention, highlighting the dominant ways in which FAIs sought to explain prison suicides.

### Ethics

A number of ethical challenges are inherent to a study of this nature. While FAIs are publicly available, they are not anonymised and often contain considerable personal information and details from individuals’ lives and deaths. We sought advice from other researchers in the field to discuss how they handled this issue and reflected carefully on balancing preservation of privacy with the desire to recognise and respect those who have died, alongside the importance of critical engagement with institutional understandings of suicide produced through FAIs. Scholars working collaboratively with families who lost loved ones to suicide in prison (Armstrong et al., 2022; Barkas et al., 2021a) used the initials of those who died as a way of navigating this tension. We have chosen to use pseudonyms in our work. While recognising the importance of engaging with families of people who have died by suicide, we were not able to do this due to issues of time, analytical approach and ethical concerns about contacting families ‘out of the blue’. In addition to pseudonymisation, we limit the use of direct quotations included from FAIs and minimise in-depth details about individuals’ backgrounds. We do not identify individual prisons within which people died. This project received ethical approval from the University of Edinburgh’s institutional ethics committee.

### Findings: Explanatory narratives of suicide in FAIs

Our analysis of the FAIs highlights ambiguity around issues of blame and responsibility, as official institutional strategies interact with broader discourses about suicide. Following our analysis of reports generated by this bureaucratic process, we wondered whether a second function of FAIs is the construction of narratives that absolve the prison of any culpability for deaths, rather than finding new ways of working or caring for incarcerated people who may be experiencing suicidal distress (see also Scraton and Chadwick, 1986). We suggest that this absolution is achieved through three dominant individualising explanatory narratives (used both on their own and in combination with one another) that we identified within FAIs: (1) mental illness, (2) social history and (3) problematic substance use. We argue that each of these narratives is underwritten by (4) an ambiguous approach to incarcerated peoples’ agency within FAIs: Incarcerated people are constructed sometimes as reliable truth tellers, while elsewhere their statements are discredited – particularly where this serves to mitigate critiques of the prison’s suicide-prevention regime.

Through these narratives, FAIs create understandings of deaths as rarely, if ever, the result of the prison system’s environment and ways of working. This paradox of blame and responsibility casts deaths as both ‘inevitable’ (e.g. no different course of action is identified that would have prevented the death) and/or ‘unforeseeable’ (e.g. the death was impossible to predict). This avoids the need to take a more critical stance on suicides in state custody – one that acknowledges the role of the prison in creating the kinds of distress that may lead to suicide. In the following sections, we consider each of these narratives in turn.

#### Mental illness

Thirty-three of the 37 FAIs included in this study mentioned mental illness as relevant to a person’s suicide. Some of these straightforwardly relate individuals’ suicidality to experiences with a range of mental health conditions, most commonly depression, anxiety and bipolar disorder. Some reports contained detailed histories of involvement with metal health and social services within and prior to incarceration, often listed chronologically, with the sheriff recounting the individual’s story through their mental health diagnoses and interactions with social support systems. An incarcerated person’s actions and character are constructed within the reports using very specific medicalised knowledge like clinical diagnoses, types of prescription medication and interactions with services. One sheriff highlights this point in relation to David’s^3^ death, noting that ‘it should not be overlooked that a significant number of prisoners have a combination of addiction and psychiatric issues’.

In addition, sheriffs made assumptions about the validity of prison staff decision-making for incarcerated people with mental health issues. In the FAI for Jim, the sheriff determines that a referral for psychiatric help would have helped the incarcerated person who died by suicide following an obvious ‘deterioration in his behaviour’. Yet, the findings suggest that because a psychiatrist had pronounced Jim was not psychotic a few days earlier, the mental health team acted appropriately. This example acknowledges that the incarcerated person’s distress was visibly increasing but claims that because normal prison procedures were followed, nothing more could have been done. An opportunity is missed here for further reflection on how existing systems of working could be improved to prevent situations like this occurring in the future. This might have included additional reflection on whether the absence of a more ‘serious’ mental health condition (such as psychosis) warrants taking no further action where there are other visible signs of distress.

Relatedly, mental illness is used as an explanation of suicide in FAIs by constructing deaths that occur in the *absence* of a specific diagnosis as ‘unforeseeable’. In one example, an individual was described as having attempted suicide by overdose, including multiple times within the prison, yet the sheriff’s report states that ‘in the absence of any active indicators of such mental illness or otherwise, which would have warranted more extreme precautions of suicide prevention, the death of [Frazer] was … realistically unavoidable’. Drawing on what Marsh (2010) refers to as the ‘compulsory ontology of pathology’ (p. 219), this *lack* of evidence of a prior mental illness is used to underline the impossibility of predicting or preventing this death by suicide, despite evidence of multiple suicide attempts. This draws on an understanding that suicide is most easily predicted and made visible by evidence or diagnoses of prior mental illnesses, reinforcing the association between psychopathology and suicidality to the exclusion of other indicators of distress – including suicide attempts.

Similarly, threats of suicide and suicidal actions are presented in the reports as contradictory to findings regarding an individual’s mental health. For example, upon his arrest, Gregor threatened to kill himself if he was remanded, yet the FAI finds that this information was not passed on to prison staff by the escort service that delivered him to prison. Despite this failure to report a newly incarcerated person’s statement of suicidal intent, the sheriff’s report emphasised that Gregor’s declaration was not necessarily a believable expression of suicidal distress, partially because ‘[Mental Health Nurse] found no evidence of an acute mental illness’. Gregor was removed from the TTM suicide-prevention strategy shortly before his death by suicide.

These examples show how FAIs mobilise mental illness to locate the causes of suicide within individual incarcerated people, tying this primarily to the presence of a psychiatric diagnosis. This effectively shifts blame away from the prison by suggesting that suicides were inevitable due to an incarcerated person’s (prior) illness. At the same time, some suicides are framed as unforeseeable due to a *lack* of formal diagnosis of mental illness – even in cases when an individual’s statements and actions indicate suicidal distress.

#### Social history

Social history was a further intersecting narrative within FAIs, sometimes mobilised in conjunction with mental illness, and sometimes alone, to explain deaths by suicide. This narrative included the breakdown of social relationships with people outside the prison. When FAIs provided a detailed life history, beyond a list of interactions with medical services, this often served to construct a fraught history of broken relationships and ‘bad’ behaviour. This effectively suggests that the ‘troubled’ background of the individual inevitably led to their suicide, reframing responsibility for the death as resulting from a combination of social factors *outside* the prison.

In the FAI for Hamilton, his death is blamed on the breakdown of his relationship with his partner, while his ‘troubled and turbulent’ childhood – including abuse, residential schooling and behavioural problems – is described in detail as further evidence to prove that his problems originated prior to, and outside of, the prison. One piece of evidence used is his suicide note, which links his death specifically to a relationship breakdown during his time in prison. This explanation is evidenced by reporting that he engaged in a challenging telephone conversation with his partner in the days prior to his suicide. In this example, we see that the FAI report locates the suicide as a problem arising from the breakdown of a relationship and a long, ‘troubled’ history, which is used to construct him as vulnerable to suicidality. This narrative represents his suicide as ‘inevitable’. The attribution of the suicide to a particular phone conversation elides the role that prisons play in disrupting or straining relationships. The narrative weaves together a number of individual factors to explain the suicide without critically engaging with the role played by the prison itself.

Several FAIs detailed similar cases, where a suicide is blamed on the breakdown of a relationship with a partner – social relationships that pre-dated the individual’s incarceration. FAIs for Gregor and David included detailed descriptions of troubled relationships prior to and during incarceration. In some of these cases, the FAIs paint deeply tragic images that place the blame for a suicide at the foot of a life of deprivation combined with a terrible phone call – or lack thereof in the case of Jim, whose FAI report notes that he died soon after trying to phone family and friends – calls that were unanswered. In Tom’s FAI, the sheriff points to his difficult experiences in local authority care following a family breakdown and even goes as far as to blame the media as a source of his distress, managing to focus responsibility anywhere but the prison. Furthermore, in summarising the details that led to Tom’s suicide, the report ignores threats to his life from other incarcerated people that are mentioned elsewhere in the report. Thus, the sheriff’s concluding statements avoid centring the role of the prison environment as a key contributor to Tom’s suicide.

Sheriffs’ engagement with social history and the breakdown of external relationships suggests an awareness of the often diffuse, networked and contextual factors that lead someone to attempt or complete suicide. However, rather than producing findings from the FAI process that might alter systems of care and support to reflect the often-fraught social contexts that incarcerated people come from, FAIs instead cast deaths in prison as both ‘inevitable’ and/or ‘unforeseeable’, ignoring how being in prison may exacerbate existing tensions within relationships and communities. While we acknowledge that distress does not begin at the prison gate, social history and relationships are not consistently engaged with for all people who die by suicide in prison – some FAI reports focus on this, while others do not. We suggest this implies social history is utilised predominantly to evidence that the prison system is not at fault, failing to acknowledge the ways in which the prison exacerbates existing social traumas and can contribute to or cause relationships to fail.

#### Drug and alcohol use

Drug and alcohol use was referenced 220 times within 26 of the reports. Along with relationship issues and mental health problems, substance use was often used to portray the character of the person who has died, providing context for their ‘troubled’ or ‘bad’ behaviour. For example, five reports noted that alcohol and drug use were indicated in previous suicide attempts and incidents of self-harm. In two reports, lack of engagement with prison substance misuse services were highlighted, and in six FAIs, a sense of hopelessness about a substance-free future was employed as an explanation of why the suicides were ‘inevitable’.

Drug use within prison was also mobilised in five reports as part of the description of behaviours that were constructed as leading to poor mental health and suicide. In the case of Ruraidh, the sheriff describes his use of illicit substances as justifying the prison staff’s inability to assess his mental health:

No further contact was thought to be required because he was unwilling to engage with the nursing team and also due to his illicit drug misuse. It was difficult to get a picture of his mental state due to these factors.

Yet in the same report, the sheriff notes that Ruraidh was placed on the TTM suicide-prevention regime upon his arrival at the prison and that there was evidence he ‘was of low mood’.

In some cases, FAIs made assumptions that an incarcerated person was using drugs even without proof or, in one case, with evidence *against* the use of drugs. The FAI report into David’s death assumes throughout that there was a link between his ‘bizarre behaviour’ and drug use, yet in the conclusion, the sheriff notes that he tested negative for illicit substances upon arrival at the prison, and none were found on him while incarcerated. David was not placed on the suicide-prevention policy because he was seen by prison officers as a ‘borderline case who did not require … close supervision’. However, the sheriff writes that staff should have erred on the side of caution by placing him under the ACT2Care programme, especially given concerns about his mental health and substance use. Curiously, the FAI report establishes drug use as relevant to the narrative despite revealing later that there was no support for this claim.

Narratives of substance use were important points of discussion within FAIs, influencing explanations of behaviour and whether to place or keep individuals on suicide-prevention programmes before their deaths. When someone has been using substances, the FAIs framed this as a poor individual choice that put them at greater risk of suicide. Substance use is used to construct a narrative which absolves the system of blame, instead placing it on the individual and their ‘bad’ choices, despite extensive research suggesting drug use is common within Scottish prisons and may indeed start within a prison context (Norman, 2022).

#### An ambiguous approach to incarcerated peoples’ agency

Throughout the FAIs, the aforementioned three explanatory narratives are mobilised to individualise deaths by suicide in Scottish prisons, yet each is underwritten by an ambiguous approach to the agency of incarcerated people. Many FAIs claim that the incarcerated individual was involved in decision-making around their care, including whether they wanted to engage with the prison’s suicide-prevention strategy. This is in line with the TTM strategy which indicates that ‘[t]he individual at risk should be involved in decisions about their own care and staff should maximise opportunities to engage with individuals regarding their care during this period’ (SPS, 2016: 7).

In the examples discussed earlier, the FAIs emphasise individual agency, seen here as present through the denial or expression of suicidal intention, as well as asking not to be placed on suicide-prevention regimes. The FAIs often emphasise that incarcerated people were asked about their suicidality but denied it. In such cases, the FAIs conclude it was therefore impossible to predict the suicide/attempt. However, this framing does not engage with the challenges that incarcerated people may face in disclosing suicidal intent in such a context, providing another way that prisons are constructed as blameless, with responsibility for disclosing suicidal ideation placed on the incarcerated person. In this sense, the FAIs recognise incarcerated people’s agency, by treating their disclosure or lack thereof as a reliable indicator of their intentions.

In cases where an incarcerated person is noted as having a mental health issue, the reports often emphasise that the person stated to mental health practitioners that they were not suicidal. The incarcerated person’s agency is subtly enrolled in a narrative of blame, with the FAI implying that they could (or should) have indicated their suicidal distress to prison staff. However, in some FAIs, where other incarcerated people have made complaints about the workings of the system (specifically suggesting the prison is culpable in the death of another incarcerated person), the sheriffs construct incarcerated people’s accounts as untrustworthy. For example, one sheriff wrote:

I am satisfied that [David] made remarks, which would be considered odd, to some of his fellow inmates. The evidence of the inmates does, however, have to be treated with care. For example, [incarcerated person 1] gave the clear impression of being an aggressive individual who had significant difficulty in refraining from using the ‘f word’. His opinion as to what steps should have been taken regarding [David] namely placed in a suicide cell, were not the result of careful consideration. [Incarcerated person 2] was clearly excitable. [Incarcerated person 3] volunteered his critical opinion of healthcare in prison. He could not recall the identity of prison officers to whom he advised [David] be seen by the mental health team.

Here, incarcerated peoples’ testimony is systematically dismissed and treated as unreliable for reasons that do not relate to their statements (including swearing and ‘excitability’). Yet their concerns appear to have been well founded because David did die by suicide.

Conversely, prison staff are generally constructed as reliable truth tellers. The same report regarding David’s death notes that his family raised a complaint about the treatment he received prior to his death. The family’s solicitor argued that there were failings in the system of care, including neglect from prison officers. However, the FAI rebuffs these claims, emphasising the experience of the prison officers involved and that they all had relevant suicide-prevention training. We identified similar patterns in other reviews – where engaging in training and being ‘experienced’ were presented as ample evidence that prison officers – and, significantly, the prison system – could not be found culpable. We argue, however, that within the institutional context of the FAI process, it is the prison system and its representatives, not incarcerated people, whose motives should be scrutinised. In the aforementioned example, incarcerated people’s agency is denied and ignored to avoid incorporating more challenging reflections on prison healthcare and the workings of the system more generally into this FAI.

FAIs also indicated that the suicide-prevention programme was sometimes implemented against the wishes of the incarcerated person. In these examples, the prison enacts logics of control and surveillance characteristic of carceral environments, demonstrating the inconsistent manner in which the policy is applied, sometimes as care and sometimes as punishment. Furthermore, despite placing individuals on suicide watch against their wishes, some still completed suicide. In one example, Grant is reported as expressing deep fears about being put on the TTM programme, with fears that the segregation unit where he would be placed was a form of punishment. This may have given Grant good reason to hide his suicidal intentions, suggesting that incarcerated people may not view suicide prevention as something to call on in moments of need but something to avoid due to the harsh consequences of disclosure.

## Discussion

We have outlined three dominant explanatory narratives in how FAIs explain suicide within Scottish prisons. These are enlisted within the FAIs to render suicides in prison as originating within the individual, rather than being produced inside or exacerbated by the prison environment. This is consistent with recent research on suicides in custody in England and Wales, which demonstrates that prison death investigations are narrowly focused on the circumstances of individual deaths, while failing to engage with the contexts of incarceration in which these deaths occur (Aitken, 2022; Tomczak et al., 2023). Narratives of suicide in the FAIs provide further examples of how ‘the inherently harmful impact of institutionalization’ is reframed as a result of damaged or deficient individuals, with blame shifted securely away from prison systems (Rylko-Bauer & Farmer, 2017: 11). Mental illness, troubled social histories and relationship breakdown and substance use were employed by FAIs in ways that construct people who have died by suicide in prison as ‘mad’ and ‘bad’. While FAI reports decontextualise prison suicides, these individualising narratives should be understood within broader cultures of punishment and othering that characterise prison environments (Barkas et al., 2021b; Rodriguez et al., 2020; Tomczak, 2018).

Our analysis indicated that the findings of many FAIs that there was ‘no fault’ in prisons where a suicide occurred may be unhelpfully affected by an ingrained culture in prison administration, staff and the court system of ‘othering’ incarcerated people, who are not treated as reliable witnesses of their environments or experiences. Indeed, FAIs are produced within a broader social context in which incarcerated people are deeply stigmatised (Spitzer, 1975; Tyler, 2020). Our findings build on the work of Buck and Tomczak (2024), which shows that even when incarcerated people are enlisted to comment on the conditions in which they are kept, this is often a one-off exception to the rule of being ignored and stigmatised. Similarly, Armstrong et al. (2021, 2022) highlight that incarcerated peoples’ accounts of their experiences are often subject to question, as was shown in the testimony of David’s fellow incarcerated people. Claims within the FAIs that incarcerated people are involved in decision-making around their inclusion in suicide-prevention practices and mental health care fail to acknowledge that the agency of people in prison is limited by the experience of incarceration, which may prevent them from feeling they can or should engage ‘openly’ with suicide-prevention policies (Gilling-McIntosh et al., 2022). Instead of engaging with the complexity of why someone might conceal suicidality, the FAIs focus on narratives that further individualise and decontextualise suicides.

Linked to this, the representation of prison suicides within FAIs as ‘inevitable’ reflects the logic of prisons as places of punishment, generating challenges in providing care, which becomes a secondary aim within this context (Armstrong et al., 2021; Barkas et al., 2021b; Mills and Kendall, 2018; Sim, 2019). We would argue – in line with the work of Mills (2020) – that prisons can be conceptualised as contexts that *invite* suicidality through various forms of violence and the inadequate provision of care (see also the study by Rylko-Bauer & Farmer, 2017). Similarly, our conclusions echo those of Sim (2018) who suggests that the prison environment ‘generates corporeal, civil and social death’ (p. 52). Within this context, it is important to consider how suicide-prevention policies may not necessarily operate as practices of ‘care’ but can be employed in ways that enact coercion and violence and deny incarcerated people’s agency. This is consistent with a recent review of the TTM strategy, which highlighted that almost half of the prisoners interviewed about their experiences of being on the TTM strategy indicated that they did not feel listened to and were not fully included in decision-making about their care (Nugent, 2018). This finding also reflects a broader question about whose perspectives are silenced or excluded from prison death reviews (Tomczak et al., 2023).^4^ One way to develop more contextual understandings of prison suicides within the FAI process would be to legitimise the perspectives of incarcerated people. As Armstrong (2020) has argued, the FAI procedure would benefit from reframing incarcerated people as valuable sources of information. Sheriffs already consult incarcerated people directly but could do so without imposing judgements on their use of language or expressions of dissatisfaction in what is likely to be an emotionally volatile experience for those who knew the person who died.

Extending the examination of the circumstances in which prison suicide occurs beyond a focus on the mental illness, social history and substance use of individual incarcerated people is in line with calls within critical suicide studies ‘to structure into our analysis of a person’s death the context of social injustice in which they lived’ (Reynolds, 2016: 170). This is also consistent with calls from researchers working on reviews of deaths in custody in England and Wales to expand the focus to systemic issues within the prison environment (Aitken, 2022; Tomczak et al., 2023). These issues include over-incarceration, understaffed and overstretched prison systems and a lack of facilities and health services (Shalev and Tomczak, 2023).

Our findings in relation to the limited nature of the FAIs are also consistent with those from a recent independent review of the responses to deaths in prison custody in Scotland, which mentions ‘the narrow focus of the FAI and the lack of broader learning from FAI findings and recommendations’ as key issues (Sinclair-Gieben et al., 2021: 13).

## Conclusion

Our analysis has demonstrated the ways in which deaths by suicide in prisons are constructed within FAIs as both ‘inevitable’ and/or ‘unforeseeable’, in effect obscuring or absolving the prison of blame and responsibility. FAI reports appear to draw on contextualising personal details when more evidence is required to effectively locate the problem that led to suicide in the individual rather than their immediate surroundings. The language used in the FAIs constructs individuals as mentally ill, socially damaged and addicted to drugs or alcohol, pointing to a system-wide acceptance of prison suicides as affecting already-marginalized people. The reporting on individual deaths mobilises these narratives to construct the individual as solely responsible for their suicide rather than considering structural-institutional issues within the prison.

We recognise that FAIs operate as highly bureaucratised processes, which are difficult to impact or change and seek to make sense of suicides after the fact – thus, an analysis of these documents produces a partial perspective on prison suicides. Engaging directly with those who have experienced suicidality, a suicide attempt or those who have witnessed a fellow incarcerated person’s suicide is necessary to develop more in-depth and nuanced understandings of prison suicide (Marzano et al., 2016). The lack of standardised procedures for writing these reports means that the amount of detail present in each review varies greatly, which constrained a more in-depth analysis. Concurrently, sheriffs’ official FAI reporting is constrained by the institutional nature of the role, and thus may not fully represent their individual beliefs about prison suicides. Yet, FAIs represent an important means through which suicide is understood, imbued with institutional power to define causes of and responses to suicide. Our analysis makes an important contribution to critical engagement with narratives of suicide in the Scottish context, with implications for understanding social scripts and practices around suicide. This is critical to exploring the problematic ways in which often already-marginalized people are further marginalized within structural-institutional-environmental settings and how this intersects with suicide. Wider calls for prison reform and abolition are ongoing (see, e.g. Davis, 2003), but ultimately these represent longer-term projects that cannot immediately address the rise of suicides in Scottish prisons. We maintain that engaging with prison environments as contributing to suicides could introduce more complexity – and meaningful action – into understandings of prison suicides.

## Data Availability

The data used in this study (Fatal Accidents and Sudden Deaths Inquiry Reports) are publicly available via the Scottish Prisons Service. In this article, we have pseudonymised the data to introduce some level of confidentiality, which may impede connecting the published reports to our data.

## Notes

1 An exception is the recent work by Sarah Armstrong and colleagues, which include statistical briefings and briefing reports that employ a thematic case analysis of FAIs.

2 In our sample, up to just over 5 years between the date of death and publication of FAI.

3 All names are pseudonyms.

4 See also the work of Armstrong et al. (2022) in relation to the exclusion of the perspectives of families in these processes.

## References

[R1] Aitken D (2022). Investigating prison suicides: The politics of independent oversight. Punishment & Society.

[R2] Armstrong S (2020). At risk of rights: Rehabilitation, sentence management and the structural violence of prison. Critical Criminology.

[R3] Armstrong S, Allan L, Allan S (2021). Nothing to See Here? Statistical Briefing on 15 Years of FAIs Into Deaths in Custody.

[R4] Armstrong S, Allan L, Cairns D (2022). Still Nothing to See Here? One Year Update on Prison Deaths and FAI Outcomes in Scotland.

[R5] Armstrong S, McGhee J (2019). Mental Health and Wellbeing of Young People in Custody: Evidence Review.

[R6] Barkas B, Allan L, Allan S (2021a). A Defective System: Case Analysis of 15 Years of Fatal Accident Inquiries After Deaths in Prison.

[R7] Barkas B, McNeill F, Casey R (2021b). Pervasive punishment in a pandemic. Probation Journal.

[R8] Bird S (2008). Changes in male suicides in Scottish prisons: 10-year study. The British Journal of Psychiatry.

[R9] Bird S (2020). Fatal accident inquiries into 83 deaths in Scottish prison custody: 2010–2013. British Journal of Psychology.

[R10] Boutcher S (2017). Private law firms and the public interest: The organisational and institutional determinants or pro bono participation, 1994-2005. Law & Social Inquiry.

[R11] Buck G, Tomczak P (2024). Prisoners regulating prisons: Voice, action, participation, and riot. Criminology and Criminal Justice.

[R12] Button M, Marsh I, Button M, Marsh I (2020). Suicide and Social Justice: Perspectives on the Politics of Suicide and Suicide Prevention.

[R13] Chandler A, Cover R, Fitzpatrick SJ (2021). Critical suicide studies, between methodology and ethics: Introduction. Health.

[R14] Davis A (2003). Are Prisons Obsolete?.

[R15] Duke K, Gleeson S, MacGregor K (2023). The risk matrix: Drug-related deaths in prisons in England and Wales, 2015-2020. Journal of Community Psychology.

[R16] Fazel S, Ramesh T, Hawton K (2017). Suicide in prisons: An international study of prevalence and contributory factors. Lancet Psychiatry.

[R17] Fitzpatrick SJ (2021). The moral and political economy of suicide prevention. Journal of Sociology.

[R18] Fleetwood J, Sandberg S, Bucerius S, Haggerty K, Berardi L (2021). Oxford University Handbook of Ethnographies of Crime and Criminal Justice.

[R19] Fraser J, Maycock M, Meek R, Woodall J (2021). Issues and Innovations in Prison Health Research.

[R20] Gilling-McIntosh L, Rees C, Kelly C (2022). Understanding the mental health needs of Scotland’s prison population. https://tinyurl.com/mwwm6j2b.

[R21] Inquiries into Fatal Accidents and Sudden Deaths (2016). (Scotland) Act 2016.

[R22] Marsh I (2010). Suicide: Foucault, History and Truth.

[R23] Marzano L, Hawton K, Rivlin A (2016). Prevention of suicidal behavior in prisons. Crisis.

[R24] Mills A, Kendall K, Mills A, Kendall K (2018). Mental Health in Prisons Critical Perspectives on Treatment and Confinement.

[R25] Mills C (2018). ‘Dead people don’t claim’: A psychopolitical autopsy of UK austerity suicides. Critical Social Policy.

[R26] Mills C, Button M, Marsh I (2020). Suicide and Social Justice: New Perspectives on the Politics of Suicide and Suicide Prevention.

[R27] Norman C (2022). A global review of prison drug smuggling routes and trends in the usage of drugs in prisons. WIREs Forensic Science.

[R28] Nugent B (2018). Evaluation of Talk to Me.

[R29] Presser L (2022). Narrative Criminology: Understanding Stories of Crime.

[R30] Presser L, Sandberg S (2019). Narrative criminology as critical criminology. Critical Criminology.

[R31] Reynolds V, White J, Marsh I, Kral MJ (2016). Critical Suicidology: Transforming Suicide Research and Prevention for the 21st Century.

[R32] Rodriguez SM, Ben-Moshe L, Rakes H (2020). Carceral protectionism and the perpetually (in) vulnerable. Criminology and Criminal Justice.

[R33] Rylko-Bauer B, Farmer P, Brady D, Button M (2017). The Oxford Handbook of the Social Science of Poverty.

[R34] ScotPHO (2023). Prisoners: Prison population.

[R35] Scottish Prison Service (2016). Talk to me strategy.

[R36] Scourfield J, Fincham B, Langer S (2012). Sociological autopsy: An integrated approach to the study of suicide in men. Social Science & Medicine.

[R37] Scraton P, Chadwick K (1986). Speaking ill of the dead: Institutionalised responses to deaths in custody. Journal of Law and Society.

[R38] Shalev S, Tomczak P (2023). Improving prisoner death investigations and promoting change in prisons: A findings and recommendations report.

[R39] Sim J, Mills A, Kendall K (2018). Mental Health in Prisons Critical Perspectives on Treatment and Confinement.

[R40] Sim J, Carlen P, Franca L (2019). Justice Alternatives.

[R41] Simms C, Scowcroft E, Isaksen M (2019). Suicide statistics report: Latest statistics for the UK and Republic of Ireland.

[R42] Sinclair-Gieben W, Loucks N, Robertson J (2021). Independent review of the response to deaths in prison custody.

[R43] Spitzer S (1975). Toward a Marxian theory of deviance. Social Problems.

[R44] Sturge G, Carthew H (2023). UK prison population statistics.

[R45] Timmermans S, Tavory I (2022). Data Analysis in Qualitative Research: Theorizing with Abductive Analysis.

[R46] Tomczak P (2018). Prison Suicide: What Happens Afterwards.

[R47] Tomczak P (2022). Highlighting ‘risky remands’ through prisoner death investigations: People with very severe mental illness transitioning from police and court custody into prison on remand. Frontiers in Psychiatry.

[R48] Tomczak P, Quinn K, Traynor C (2023). (Re)constructing prisoner death investigations: A case study of suicide investigations from England and Wales. Law & Social Inquiry.

[R49] Tweed EL, Gounari X, Graham L (2021). Mental wellbeing among people in prison in Scotland: An analysis of repeat cross-sectional surveys. Journal of Mental Health.

[R50] Tyler I (2020). Stigma: The Machinery of Inequality.

[R51] White J (2017). What can critical suicidology do?. Death Studies.

[R52] White J, Marsh I, Kral MJ (2016). Critical Suicidology: Transforming Suicide Research and Prevention for the 21st Century.

[R53] Wilson M, Johnston L, Walker L (2020). ‘It was like an animal in pain’: Institutional thoughtlessness and experiences of bereavement in prison. Criminology and Criminal Justice.

